# Joint Design of Transmit Waveforms for Object Tracking in Coexisting Multimodal Sensing Systems

**DOI:** 10.3390/s19081753

**Published:** 2019-04-12

**Authors:** John S. Kota, Antonia Papandreou-Suppappola

**Affiliations:** 1 Systems and Technology Research, Sensors & Signal Processing Group, Woburn, MA 01801, USA; john.kota@stresearch.com; 2 School of Electrical, Computer and Energy Engineering, Arizona State University, Tempe, AZ 85287-5706, USA

**Keywords:** multiple object tracking, waveform design for active sensing, spectrum sharing, pulse–Doppler radar, multiuser wireless communications, multiobjective optimization

## Abstract

We examine a multiple object tracking problem by jointly optimizing the transmit waveforms used in a multimodal system. Coexisting sensors in this system were assumed to share the same spectrum. Depending on the application, a system can include radars tracking multiple targets or multiuser wireless communications and a radar tracking both multiple messages and a target. The proposed spectral coexistence approach was based on designing all transmit waveforms to have the same time-varying phase function while optimizing desirable performance metrics. Considering the scenario of tracking a target with a pulse–Doppler radar and multiple user messages, two signaling schemes were proposed after selecting the waveform parameters to first minimize multiple access interference. The first scheme is based on system interference minimization, whereas the second scheme explores the multiobjective optimization tradeoff between system interference and object parameter estimation error. Simulations are provided to demonstrate the performance tradeoffs due to different system requirements.

## 1. Introduction

A multimodal system can be used to provide significant performance improvements in tracking multiple objects by integrating information from asymmetric sensors. When tracking multiple but similar objects, receiver processing at a given time requires estimation of information that the objects have in common, such as object states, object label and time-varying cardinality. Approaches used for this problem include random finite set methods with probability hypothesis density filtering, multi-Bernoulli or labeled multi-Bernoulli filtering, and more recently, nonparametric Bayesian methods to model state priors [1,2,3,4,5,6,7]. When tracking objects with different types of unknown information, applicable sensing modalities can be appropriately designed to increase overall system performance. In some cases, however, performance can suffer from operational conditions that result in system interference. For example, both target position and user communication messages must be estimated in a system with both radar and wireless communication modalities [8,9]. However, if the modalities operate in adjacent bands, the ever-increasing demands on the operational spectrum can cause growing levels of interference. Currently, spectrum congestion has affected weather radars [10], airport surveillance radars [11], remote sensing systems [12], and military radar and communications systems [13,14,15,16]. A recent approach to deal with spectrum congestion is system spectrum sharing while designing system parameters to reduce interference.

Various methods have been considered in designing the transmit waveforms of different systems that share spectra. Codesign methods construct the transmit waveform of one system to minimize strong interference caused by other systems [17,18,19,20,21]. Coexistence methods, on the other hand, design a joint transmit waveform for multiple spectrum sharing systems. Various such approaches were developed for coexisting radar and communications systems [18,22,23,24,25,26,27,28,29]. For example, both systems shared orthogonal frequency division multiplexing signaling in References [22,23] and linear frequency-modulated chirp signaling in References [24,25,26]. In Reference [27], radar and communications coexistence was achieved using joint channel estimation and adaptive parameter optimization, whereas maximization of the combined mutual information was used in Reference [28]. Note that coexistence has also been considered in heterogeneous systems, such as narrowband and ultrawideband networks and wireless networks [30,31,32,33,34,35,36,37].

In this manuscript, we propose a new signaling scheme for use in tracking a moving target and a time-varying number of user messages using a coexisting radar and multiuser wireless communications system. The scheme adapts a transmit waveform with the same nonlinear phase function and with parameters that are selected to optimize performance metrics under the constraint of given system criteria. Such criteria include fixed range resolution for estimating target position and fixed gross data rates for communications transmission. The performance metrics considered are interference between users, interference between systems, and error in estimating object states. The transmit waveforms of the communications users were first designed to reduce multiple access user interference. Then, the transmit waveform of the radar was selected so that the interference between the two systems is minimized. A second waveform design approach was considered that uses multiobjective (Pareto) optimization to jointly minimize overall system interference and mean-square error of object state estimation. Tradeoffs in system performance were demonstrated using a pulse–Doppler radar that shares the same spectrum with a multiple phase-shift keying communications system.

The rest of the paper is organized as follows. In Section 2, we describe the common transmit waveform used by the two systems. We also provide the waveform parameter selection criteria for maximizing correlation under various optimization constraints. The processing of the system received waveforms is summarized in Section 3, and system performance optimization criteria are provided in Section 4. Finally, Section 5 provides simulations to demonstrate the proposed joint waveform design approach.

## 2. Orthogonal Waveforms with Nonlinear Phase Function

A general form of a time-varying (TV) waveform can be given in terms of a TV amplitude function a(t) and a TV phase function $\xi (t/t_{r})$ as:

(1) $$s\left(t\right)=a\left(t\right)\;e^{j2\pi b\;\xi (t/t_{r})}\;,\;t\in \Xi_{t},$$

where *b* ∈ R is the frequency-modulation (FM) rate, $\Xi_{t}$ is the range of the time values *t*, and $t_{r}$ > 0 is a normalization time constant. An orthogonal TV waveform can be obtained from Equation (1) under certain constraints [38]. The first constraint is that the TV amplitude function is given by:

(2) $$a\left(t\right)=\sqrt{\left|\nu (t)\right|}={\left|\frac{d}{dt}\xi \left(t/t_{r}\right)\right|}^{1/2},$$

where ν(t) is the waveform’s instantaneous frequency, obtained as the derivative of the phase function. The second constraint is that the TV phase function $\xi (t/t_{r})$ must be a monotonic function whose range is γ = $\xi (t/t_{r})$ ∈ R. Then, assuming that both constraints hold, we define the *m*th TV orthogonal waveform, *m* ∈ N, with FM rate $b_{m}$, as $s_{m}\left(t\right)$ = $\sqrt{\left|\nu (t)\right|}\;e^{j2\pi b_{m}\;\xi \left(t/t_{r}\right)}$, where the inverse function of $\xi (t/t_{r})$ exists with range $\Xi_{t}$, $\xi (t/t_{r})$ ∈ R, $t_{r}$ > 0, and ν(t) = $d/dt(\xi \left(t/t_{r}\right))$ in Equation (2). It can then be shown that the correlation of the two orthogonal signals $s_{m}\left(t;b_{m}\right)$ and $s_{l}\left(t;b_{l}\right)$ is given by:

(3) $$\int_{\Xi_{t}}s_{m}\left(t;b_{m}\right)\;s_{l}^{*}\left(t;b_{l}\right)\;dt=\int_{\Xi_{t}}\left|\nu \left(t\right)\right|\;e^{j2\pi \left(b_{m}-b_{l}\right)\;\xi \left(t/t_{r}\right)}\;dt=\int_{R}e^{j2\pi (b_{m}-b_{l})\;\gamma }\;d\gamma =\delta \left(b_{m}-b_{l}\right)\;,$$

where δ(·) is the Dirac delta function and m,l ∈ N.

An example of a TV waveform that satisfies Equation (3) is the orthogonal linear frequency-modulated (OLFM) waveform that is specifically defined with quadratic phase function $\xi (t/t_{r})$ = $\mathrm{sgn}\left(t\right)|t/t_{r}{|}^{2}$, *t* ∈ R, where sgn(t) is ±1 depending on the sign of *t*, and TV amplitude amplitude function given by a(t) = $\sqrt{2|t|/t_{r}^{2}}$ in Equation (2). When the waveforms are used in practice with finite duration, orthogonality no longer holds. However, approximate orthogonality conditions can be obtained by minimizing the correlation between two finite duration waveforms. Specifically, the *m*th OLFM waveform, *m* = 1,…,M, with finite duration *T* and FM rate $b_{m}$ is given by:

(4) $$s_{m}\left(t;b_{m}\right)=\sqrt{2\;t}\;e^{j2\pi b_{m}\;t^{2}},\;t\in \left(0,T\right)\;.$$

Here, $t_{r}$ = 1 without loss of generality. For approximate orthogonality, the absolute correlation between any two finite duration OLFM waveforms, $s_{m}\left(t;b_{m}\right)$ and $s_{l}\left(t;b_{l}\right)$, m,l = 1,…,M, must satisfy:

(5) $$\Gamma_{m,l}=\left|\int_{0}^{T}2\;t\;e^{j2\pi \left(b_{m}-b_{l}\right)t^{2}}\;dt\right|=\left|\int_{0}^{T^{2}}\;e^{j2\pi (b_{m}-b_{l})\tau }d\tau \right|=|T^{2}\;\sin c\left(\left(b_{m}-b_{l}\right)\;T^{2}\right)|=\delta \left[m-l\right],$$

where sinc(x)≜sin(πx)/πx and δ[m] is the Kronecker delta function. Thus, the condition in Equation (5) holds, provided the FM rates, $b_{m}$ and $b_{l}$, of the OLFM waveforms are selected to satisfy:

(6) $$b_{m}-b_{l}=\frac{\ell }{T^{2}},\;\ell =0,\pm 1,\pm 2,\ldots$$

**Figure 1:**
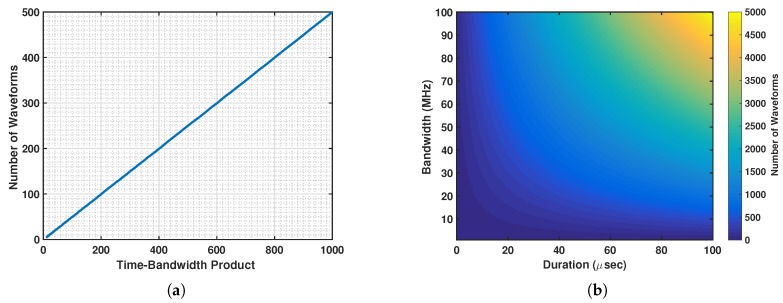
Number *M* of approximate orthogonal linear frequency-modulated (OLFM) waveforms in Equation (4) for varying (**a**) time–bandwidth product TB; and (**b**) both duration *T* and bandwidth *B*.

If we only select positive FM rates, $b_{m}$ ∈ $R^{+}$, then Equation (6) simplifies to $b_{m}-b_{l}$ = $\ell /T^{2}$, *ℓ* ∈ $N^{+}$, for *m* > *l*. Note that the values of the FM rates are constrained by the waveform bandwidth *B*, as the instantaneous frequency of the *m*th OLFM waveform in Equation (4) is given by ν(t) = $2\;b_{m}\;t$, *t* ∈ (0,T). Thus, the largest possible FM value $b_{M}$ of the OLFM waveform must satisfy *B* = $2\;b_{M}\;T$. The maximum number *M* of approximate OLFM waveforms that can be assigned a unique positive FM rate that satisfy Equation (5) is thus given by *M* = ⌊TB/2⌋. The variation of this number as a function of the OLFM waveform time–bandwidth product, and as a function of both the OLFM waveform duration *T* and bandwidth *B* is demonstrated in Figure 1a,b, respectively.

## 3. Spectrum Sharing Radar and Communications Systems

### 3.1. Common Transmit Waveform of Coexisting Systems

We considered two coexisting systems that share a spectrum in the S band. The first system is a monostatic pulse–Doppler radar used to determine the range and velocity of a nonfluctuating deterministic (Swerling-0 model) target. The second one is a wireless multiuser (MU) communications system used to transmit multiple symbols for *M* users over each pulse repetition interval (PRI) of the radar. The receivers of the two systems are assumed to be collocated.

We assumed that both systems use a transmit waveform with the same time-varying amplitude and phase functions. In particular, both systems use the OLFM waveform in Equation (4) with fixed bandwidth *B* but with varying FM rate *b* and duration *T*. We denoted the transmit OLFM waveform for the radar as $s_{r}\left(t\right)$ = $s_{r}\left(t;b_{r},T_{r}\right)$ and for the *m*th communications user as $s_{c,m}\left(t\right)$ = $s_{c,m}\left(t;b_{m},T_{c}\right)$, *m* = 1,…,M. The radar parameter set, with cardinality 2, is given by:

(7) $$\Psi_{r}=\left\{T_{r},\;\;b_{r}\right\}\;.$$

Assuming all *M* communications users have OLFM waveforms with the same finite duration $T_{c}$ but unique FM rates, then the MU communications parameter set, with cardinality (M+1), is given by:

(8) $$\Psi_{c}=\left\{T_{c},\;\;b_{1},\;\;b_{2},\;\;\ldots ,\;\;b_{M}\right\}\;.$$

Thus, overall, there are (M+3) parameters that must be designed at each coherent processing interval (CPI) of the radar. Our proposed coexisting transmit waveform scheme (CoWS) design is based on optimizing waveform-dependent system performance metrics; such metrics include system-specific ones, including multiple access interference (MAI), gross bit rate bit rate, transmission bit-error rate (BER), and range resolution, and mean-squared error (MSE) of parameter estimation, as well as joint metrics, such as interference between the two systems (see Section 4). We first provide some background on the processing of the received waveforms for each system that is required when optimizing the waveform-dependent performance metrics.

### 3.2. Pulse–Doppler Radar Receiver

#### 3.2.1. Radar Received Waveform

The pulse–Doppler radar was assumed to transmit *K* pulses over the CPI. The received baseband signal from the *k*th transmitted pulse, *k* = 1,…,K, using a sampling period $T_{s}$ and a PRI $T_{\mathrm{PRI}}$, is:

(9) $$z_{r,k}\left[n\right]=x_{r,k}\left[n\right]+x_{c,k}\left[n\right]+w_{k}\left[n\right],\;n=1,\ldots ,N_{s}\;,$$

where $x_{c,k}\left[n\right]$ is the observed communications signal on the collocated receiver, $w_{k}\left[n\right]$ is additive white Gaussian noise (AWGN), $N_{s}$ = $\left\lfloor T_{\mathrm{PRI}}/T_{s}\right\rfloor$ is the number of samples, and the radar return is:

(10) $$x_{r,k}\left[n\right]=\sqrt{P_{r}}\;s_{r}\left(nT_{s}-\tau_{0}-kT_{\mathrm{PRI}}\right)\;e^{-j2\pi \nu_{0}\;k\;T_{\mathrm{PRI}}},\;n=1,\ldots ,N_{s}\;.$$

Here, $P_{r}$ is the power of the radar return signal and $s_{r}\left(t\right)$ is the radar transmitted waveform. Over the CPI, both the time shift $\tau_{0}$ and the frequency shift $\nu_{0}$ are assumed to be constant. Observing all *K* transmitted pulses over the CPI, the overall radar received waveform is given by:

(11) $$z_{r,\mathrm{CPI}}\left[n\right]=\sum_{k=1}^{K}z_{r,k}\left[n\right],\;n=1,\ldots ,N_{s}\;.$$

The overall waveform over the CPI in Equation (11) can also be expressed in vector form $z_{r,\mathrm{CPI}}$ ∈ $C^{N_{s}\;K\times 1}$ as $z_{r,\mathrm{CPI}}$ = $\mathrm{vect}(Z_{r})$, where $\mathrm{vect}(Z_{r})$ denotes vectorization of matrix $Z_{r}$ by stacking the matrix columns into a single column vector. The matrix $Z_{r}\in C^{N_{s}\times K}$ is given by:

(12) $$Z_{r}=\left[z_{r,1}\;\;\ldots \;\;z_{r,K}\right]=\sqrt{P_{r}}\;S_{r}\left(\tau_{0}\right)\;D^{H}\left(\nu_{0}\right)+X_{c}+W,$$

where $z_{r,k}$ = ${\left[z_{r,k}\left[1\right]\ldots z_{r,k}\left[N_{s}\right]\right]}^{T}$, $z_{r,k}$ ∈ $C^{N_{s}\times 1}$, T and H denote vector transpose and Hermitian transpose, respectively, $S_{r}\left(\tau_{0}\right)$ ∈ $C^{N_{s}\times K}$ is $S_{r}\left(\tau_{0}\right)$ = $\left[s_{r}\left(\tau_{0};1\right)\ldots s_{r}\left(\tau_{0};K\right)\right]$, and $s_{r}\left(\tau_{0};k\right)$ ∈ $C^{N_{s}\times 1}$ is:

(13) $$s_{r}\left(\tau_{0};k\right)={\left[s_{r}\left(T_{s}-\tau_{0}-kT_{\mathrm{PRI}}\right)\;\;\ldots \;\;s_{r}\left(N_{s}T_{s}-\tau_{0}-kT_{\mathrm{PRI}}\right)\right]}^{T}\;.$$

The other matrices in Equation (12) include the diagonal Doppler matrix $D(\nu_{0})$ ∈ $C^{K\times K}$ given by $D(\nu_{0})$ = $\mathrm{diag}(d\left(\nu_{0}\right))$, with diagonal entries $d(\nu_{0})$ = $\left[e^{j2\pi \nu_{0}T_{\mathrm{PRI}}}\;\ldots \;e^{j2\pi K\nu_{0}T_{\mathrm{PRI}}}\right]$, $d(\nu_{0})$ ∈ $C^{1\times K}$; matrix $X_{c}$ ∈ $C^{N_{s}\times K}$ given by $X_{c}$ = $\left[x_{c,1}\;\ldots \;x_{c,K}\right]$ with $x_{c,k}$ ∈ $C^{N_{s}\times 1}$, $x_{c,k}$ = ${\left[x_{c,k}\left[1\right]\;\ldots \;x_{c,k}\left[N_{s}\right]\right]}^{T}$; and matrix W ∈ $C^{N_{s}\times K}$ given by W = $\left[w_{1}\;\ldots \;w_{K}\right]$ with $w_{k}$ ∈ $C^{N_{s}\times 1}$, $w_{k}$ = ${\left[w_{k}\left[1\right]\;\ldots \;w_{k}\left[N_{s}\right]\right]}^{T}$. Note that the columns of matrix $X_{c}$ correspond to the communications symbols of all the users over each PRI.

#### 3.2.2. Radar Receiver Processing

The processing at the radar receiver requires the estimation of the target range and velocity at each time step. The overall received waveform at the receiver must be correlated with all possible time-delayed and frequency-shifted versions of the transmitted signal. Due to the pulse–Doppler processing, range is estimated from slow-time processing that involves the PRI time step *k*, whereas velocity is estimated from fast-time processing that involves the time sample *n*. The correlation matrix $A_{r}$ ∈ $C^{K\times N_{\tau }}$ over all possible $N_{\tau }$ time delays (or range bins) is first formed as:

$$A_{r}=Z_{r}^{H}\;S_{\tau }\;,$$

where $Z_{r}$ is the received signal matrix in Equation (12), $S_{\tau }$ ∈ $C^{N_{s}\times N_{\tau }}$ is $S_{\tau }$ = $\left[s_{r}\left(\tau_{1};k\right)\;\ldots \;s_{r}\left(\tau_{N_{\tau }};k\right)\right]$, $s_{r}\left(\tau_{\ell };k\right)$ is given in Equation (13), and $\tau_{\ell }$, *ℓ* = $1,\ldots ,N_{\tau }$ denotes the *ℓ*th time-delay or range bin. The domain $\left[T_{r},T_{\mathrm{PRI}}\right]$ of $\tau_{\ell }$ represents the domain of unambiguous target returns, where $T_{r}$ is the duration of the transmit radar signal $s_{r}\left(t\right)$. The (ℓ,m)th element $a_{\ell ,k}$, *ℓ* = $1,\ldots ,N_{\tau }$, *k* = 1,…,K, of $A_{r}$ is given by:

(14) $$a_{\ell ,k}=\sum_{n=1}^{N_{s}}z_{r,k}\left[n\right]\;s_{r}^{*}\left(nT_{s}-\tau_{\ell }-kT_{\mathrm{PRI}}\right)=z_{r,k}^{H}s_{r}\left(\tau_{\ell };k\right)\;.$$

In Equation (14), the target range can be estimated when the time-shifted transmitted signal is correlated with the received signal at the *k*th PRI in Equation (10). In Equation (14), the vector $z_{r,k}$ can also be given by:

(15) $$z_{r,k}=\sqrt{P_{r}}\;s_{r}\left(\tau_{0};k\right)\;e^{-j2\pi \nu_{0}\;k\;T_{\mathrm{PRI}}}+x_{c,k}+w_{k},\;k=1,\ldots ,K\;,$$

where $s_{r}\left(\tau_{0};k\right)$ is defined in Equation (13).

After *K* pulses are received, the discrete-time Fourier transform (DTFT) is computed across the rows of A to estimate the $N_{\nu }$ > *K* frequency shifts. Using the DTFT matrix $F_{\nu }$ ∈ $C^{N_{\nu }\times K}$, $F_{\nu }$ = $\left[\phi_{1}\;\ldots \;\phi_{K}\right]$, where $\phi_{k}$ ∈ $C^{N_{\nu }\times 1}$, $\phi_{k}$ = ${\left[e^{j2\pi \nu_{1}kT_{\mathrm{PRI}}}\;\ldots \;e^{j2\pi \nu_{N_{\nu }}kT_{\mathrm{PRI}}}\right]}^{H}$, *k* = 1,…,K, the overall pulse–Doppler output matrix $Y_{r}$ ∈ $C^{N_{\nu }\times N_{\tau }}$ is given by:

(16) $$Y_{r}=F_{\nu }A_{r}=F_{\nu }\;Z_{r}^{H}\;S_{\tau }\;.$$

Using Equation (12), the pulse–Doppler output in Equation (16) can be written as:

(17) $$Y_{r}=\sqrt{P_{r}}\;F_{\nu }\;D\left(\nu_{0}\right)\;S_{r}^{H}\left(\tau_{0}\right)\;S_{\tau }+F_{\nu }{\left(X_{c}+W\right)}^{H}S_{\tau }=\sqrt{P_{r}}\;X_{r,\nu ,\tau }+{\tilde{X}}_{c}+\tilde{W}\;.$$

The radar processed output, with the noise and communications interference component, is given by $X_{r,\nu ,\tau }$ = $F_{\nu }\;D\left(\nu_{0}\right)\;S_{r}^{H}\left(\tau_{0}\right)\;S_{\tau }$ and has the form of the ambiguity function of the transmitted signal. This follows from its dependence on $S_{r}\left(\tau_{0}\right)$ = $\left[s_{r}\left(\tau_{0};1\right)\ldots s_{r}\left(\tau_{0};K\right)\right]$ and $S_{\tau }$ = $\left[s_{r}\left(\tau_{1};k\right)\;\ldots \;s_{r}\left(\tau_{N_{\tau }};k\right)\right]$, where $s_{r}\left(\tau_{\ell };k\right)$, *ℓ* = $0,1,\ldots ,N_{\tau }$, is defined in terms of the radar transmit waveform in Equation (13). When this transmit waveform is selected to follow the CoWS, the radar processed output directly depends on the radar parameter set $\Psi_{r}$ = $\left\{T_{r},\;b_{r}\right\}$ in Equation (7), and it is given by:

(18) $$X_{r,\nu ,\tau }\left(\Psi_{r}\right)=F_{\nu }\;D\left(\nu_{0}\right)\;S_{r}^{H}\left(\tau_{0};\Psi_{r}\right)\;S_{\tau }\left(\Psi_{r}\right)\;.$$

From Equation (17), the interference from the communications system is given by:

(19) $${\tilde{X}}_{c}\left(\Psi_{c},\Psi_{r}\right)=F_{\nu }\;X_{c}^{H}\left(\Psi_{c}\right)\;S_{\tau }\left(\Psi_{r}\right)\;.$$

Note that it depends on both the radar parameter set, due to the term $S_{\tau }\left(\Psi_{r}\right)$ and the parameters $\Psi_{c}$ in Equation (8) of all the users transmitting, due to the term $X_{c}$ that depends on $x_{c,k}\left[n\right]$ in Equation (9). The noise contribution in Equation (17) is given by $\tilde{W}$ = $F_{\nu }\;W^{H}\;S_{\tau }\left(\Psi_{r}\right)$.

### 3.3. Wireless Multiuser Communications Receiver

#### 3.3.1. Communications Received Waveforms

For a wireless communications system with *M* users, the transmit waveform of the *m*th user, *m* = 1,…,M, $s_{c,m}\left(t\right)$, is assumed to have duration $T_{c}$. Without loss of generality, and before transmission, we assumed that the waveform is modulated using multiple phase shift keying (PSK) of order *P* (*P*-PSK). The modulated waveform is given by $s_{c,m}\left(t\right)e^{j2\pi (p_{m}-1)/P}$, where $p_{m}$ = 1,…,P, is the modulation phase shift index used by the *m*th user. Each user could transmit up to $\log_{2}\left(P\right)$ bits of information per symbol over the duration of the waveform and *Q* = $\left\lfloor T_{\mathrm{PRI}}/T_{c}\right\rfloor$ symbols over one PRI of the radar system.

Considering the *k*th PRI, and assuming a sampling period $T_{s}$, the continuous train of transmitted symbols by the *m*th user is given by:

(20) $$s_{c,m,k}\left[n\right]=\sqrt{P_{c}}\;\sum_{q=0}^{Q-1}s_{c,m}\left(nT_{s}-qT_{c}-kT_{\mathrm{PRI}}\right)\;e^{j2\pi (p_{m,q,k}-1)/P},\;n=1,\ldots ,N_{s}\;.$$

Here, $P_{c}$ is the power of the return signal, and $p_{m,q,k}$, for $p_{m,q,k}$ = 1,2,…,P, is the phase shift index of the *P*-PSK constellation point representing the *q*th symbol, *q* = 0,…,Q−1, of the *m*th user, *m* = 1,…,M, in the *k*th PRI, *k* = 1,…,K.

Assuming an AWGN communications channel, the overall signal received at the communications receiver by all users during the *k*th PRI is given by:

(21) $$z_{c,k}\left[n\right]=x_{c,k}\left[n\right]+x_{r,k}\left[n\right]+w_{k}\left[n\right]=\sum_{m=1}^{M}s_{c,m,k}\left[n\right]+x_{r,k}\left[n\right]+w_{k}\left[n\right]\;,\;n=1,\ldots ,N_{s}\;,$$

where $s_{c,m,k}\left[n\right]$ is provided in Equation (20). Note that signal terms $x_{c,k}\left[n\right]$, $x_{r,k}\left[n\right]$, and $w_{k}\left[n\right]$ are as provided in the radar return in Equations (9) and (10).

#### 3.3.2. Communications Receiver Processing

At the receiver of the communications system, the *q*th symbol, *q* = 0,…,Q−1, that is transmitted by the *m*th user, *m* = 1,…,M, during the *k*th PRI, *k* = 1,…,K, can be estimated by:

(22) $${\hat{p}}_{m,q,k}=\arg\max_{\begin{array}{c} p_{m,q,k}=1,\ldots ,P \end{array}}\;\left\{\Lambda_{m,q,k}\right\}\;.$$

This optimization results in the index of the phase shift that yields the maximum correlation given by:

(23) Λm,q,k=Re∑n=qnc(q+1)nczc,k[n]sc,m(nTs−qTc−kTPRI)e−j2π(pm,q,k−1)/P.

Note that this correlation term includes not only the noisy transmitted waveform from the *m*th user, but it also assumes the presence of the radar return. From Equation (23), the correlation is shown to depend on the transmit waveform $s_{c,m,k}\left[n\right]$ and thus $s_{c,m}\left[n\right]$ in Equation (20). Using CoWS, the *m*th user’s communication signal depends on the parameter set $\Psi_{c}$ = $\left\{b_{m},\;T_{c}\right\}$ in Equation (8) (for one user); thus, $s_{c,m}\left[n\right]$ = $s_{c,m}\left[n;b_{m},\;T_{c}\right]$. In addition, the signal term $z_{c,k}\left[n\right]$ in Equation (23) depends on $x_{r,k}\left[n\right]$ in Equation (21), which is defined in Equation (9); this radar interference term causes the correlation in Equation (23) to also depend on the radar parameter set $\Psi_{r}$ = $\left\{b_{r},\;T_{r}\right\}$ in Equation (7) since $x_{r,k}\left[n\right]$ = $x_{r,k}\left[n;b_{r},\;T_{r}\right]$. As a result, in order to obtain a better estimate of $p_{m,q,k}$ in Equation (22), the correlation has be optimized over all possible radar and communication parameters. This dependence is shown as:

(24) Λm,q,k(Ψc,Ψr)=Re∑n=qnc(q+1)nczc,k[n;Ψc,Ψr]sc,m(nTs−qTc−kTPRI;Ψc)e−j2π(pm,q,k−1)/P.

Note that in Equation (24), it is already assumed that the receiver has determined that the received waveform is from the *m*th user.

## 4. Waveform-Dependent Performance Optimization Methods

Our main objective was the design of a transmit waveform that is common for coexisting radar and MU communications systems under some performance metric constraints. As multiple studies have demonstrated over the last decades, each system considered separately encounters numerous performance tradeoffs when designing their transmit waveform. Considering the pulse–Doppler radar in Section 3.2, its transmit waveform can be selected, for example, to maximize the radar’s range and range–rate resolution in order to reduce the MSE of the estimated position and velocity of a target when moving in a noisy environment. The transmit waveform for a single user in a wireless communications system must be selected to maximize the gross bit rate while reducing the BER performance. When multiple users are transmitting, each user’s transmit waveform must also be designed to reduce interference between each other. As a result, performance tradeoffs must be considered between desirable gross bit rate, BER and MAI. While the aforementioned performance metrics were system-specific, other metrics, such as one system acting as interference to the other system, affect both systems.

It is unrealistic to expect that one common transmit waveform can optimize system-specific and common performance metrics for both systems. As a preprocessing step, however, we started our waveform design by making use of some established results. In particular, it has been well-established that radar transmit waveforms with quadratic time-varying phase function improve range resolution in radars [39]. We have also recently demonstrated that these waveforms improve radar MSE performance under low signal-to-interference-plus-noise ratio (SINR), and in particular, in the presence of high communications interference [17,21]. As a result, we first concentrated on a performance metric that is specific only to MU communications systems: We applied waveforms with quadratic time-varying phase function to reduce MAI. We can then extend the CoWS design to optimize common system performance metrics.

### 4.1. MAI Mitigation in MU Communications Systems

Following the proposed CoWS design, the *m*th commications user is assigned the OLFM waveform $s_{m}\left(t\right)$ = $s_{c,m}\left(t\right)$ in Equation (4) with duration *T* = $T_{c}$ and a unique FM rate $b_{m}$ that must satisfy:

(25) $$b_{m}=\frac{B}{2T_{c}}-\left(\frac{M-m}{T_{c}^{2}}\right)\;,\;m=1,\ldots ,M\;,$$

for minimum MAI, following Equation (6) [21,40], for a given bandwidth *B*. The maximum number of users transmitting at the same time with an assigned unique FM rate is *M* = $\left\lfloor T_{c}B/2\right\rfloor$ (see Section 2). This number increases with the time–bandwidth product $T_{c}B$, as shown in Figure 1a. Figure 1b also demonstrates that the number increases with both the pulse duration and the allocated bandwidth.

The gross bit rate *R* affects the user’s BER performance since, for a fixed PSK modulation order *P*, the symbol duration is affected following the relation:

(26) $$R=\frac{1}{T_{c}}\log_{2}\left(P\right)\;.$$

**Figure 2:**
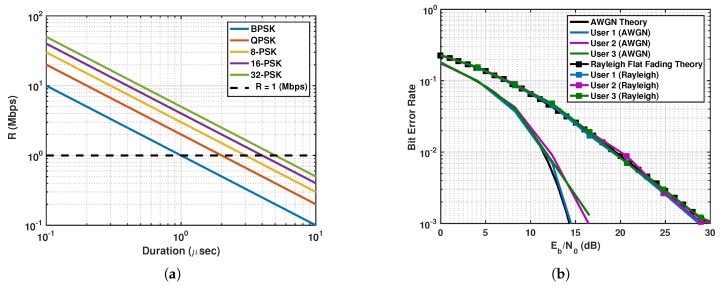
(**a**) Feasible gross bit rate *R* as a function of symbol duration $T_{c}$ = *T* for phase shift keying (PSK) modulation orders *P* = 2,4,8,16,32. (**b**) Bit-error rate (BER) performance for varying signal-to-noise ratio (SNR) for three users transmitting over an additive white Gaussian noise (AWGN) channel and a Rayleigh fading channel using the designed OLFM waveform with 16-PSK modulation.

This is demonstrated for an OLFM waveform in Equations (4) and (20) in Figure 2a for varying *P* and symbol duration $T_{c}$. As can be seen, for a given modulation order, the gross bit rate increases as the duration decreases. However, for a fixed bandwidth, when the duration decreases, the maximum number of users that can transmit over the communication channel also decreases. The BER performance for a varying signal-to-noise ratio (SNR) is shown in Figure 2b for an MU AWGN and Rayleigh fading channels. Three users are transmitting using the designed CoWS with fixed bandwidth, fixed duration, 16-PSK, and FM rates as in Equation (25). Note that for the AWGN channel, the estimated symbols needed for computing BER can be obtained as in Equations (22) and (24) but without the presence of the radar interference. The SNR is computed as $E_{b}/N_{0}$, where $E_{b}$ is the energy per bit and $N_{0}$ is the variance of the AWGN samples. Note that the BER is computed theoretically and also obtained using Monte Carlo simulations in Figure 2a,b. For our proposed coexisting systems, we assumed that the MU communications system has two main objectives. The first is to maintain a desirable level of gross bit rate *R* for a given multiple PSK modulation order *P* in Equation (26); this constrains the symbol duration $T_{c}$. The second one is a minimum level of MAI; for a given bandwidth *B* and using OLFM waveforms, this constrains the selection of the FM rates to satisfy Equation (25). Thus, the optimal communications parameter set for a desirable level of gross bit rate and minimum MAI results in the OLFM waveform parameter set $\Psi_{c}$ given by Equation (7).

If we assume that the actual number of users that are transmitting is $N_{c}$ ≤ *M*, then the FM rate assignment in Equation (25) can be revised to further minimize MAI. In particular, assuming that the symbol transmission between users is synchronized in time, we can select the $N_{c}$ FM rate values that correspond to the OLFM waveforms that yield the minimum correlation. In particular, the optimal communications parameter set is:

(27) $$\Psi_{c}=\left\{T_{c},\;{\hat{b}}_{1},\;\ldots ,\;{\hat{b}}_{N_{c}}\;\right\}=\arg\min_{\begin{array}{c} N_{c}\;\mathrm{values}\\ b\in \Theta_{c} \end{array}}\left\{\sum_{m,l,m\neq l}{\left|\int_{0}^{T_{c}}s_{c,m}\left(t;b_{m}\right)\;s_{c,l}^{*}\left(t;b_{l}\right)\;dt\right|}^{2}\right\}\;,$$

where $\Theta_{c}$ = $\left\{b_{1},\;b_{2},\;\ldots ,\;b_{M}\right\}$ is a set of possible FM rates that satisfy the relation in Equation (6), and the OLFM waveform $s_{c,m}\left(t;b_{m}\right)$ is given in Equation (4). Note that $T_{c}$ is still obtained from the desirable gross bit rate for a given PSK modulation *P*. Note that even when $N_{c}$ increases and this becomes a combinatorics optimization problem, it can be solved using the simulated annealing stochastic optimization method [41], which allows for non-optimum points to avoid local minima values.

### 4.2. Approach I: Coexistence Waveform Design Approach by Minimizing System Interference

We designed the optimal radar parameter set in Equation (7) by minimizing the interference between the radar and the MU communications system. This requires that the radar have some prior knowledge on the transmit parameter set of the collocated communications system. As expected, the performance of the CoWS design is improved as additional prior information on the communications transmit waveforms becomes available the radar.

In the following sections, we assume that the coexisting radar and MU communications system are using the CoWS design based on the OLFM waveforms with FM rates as in Equation (25) and shared bandwidth *B*.

#### 4.2.1. Approach I-A: Radar Has Knowledge of Symbol Duration of Communications Users

**Figure 3:**
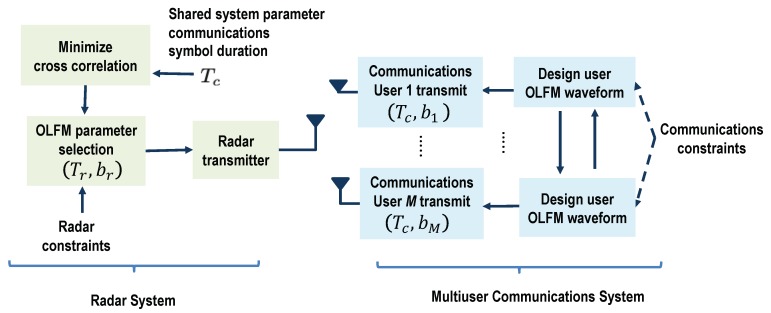
Block diagram overview of coexistence waveform design Approach I-A: The radar receiver has knowledge of the OLFM waveform duration, $T_{c}$, that is common to all *M* communications users.

We assumed that the only prior knowledge the radar has is the fixed symbol duration $T_{c}$ used by all the communications users. This is demonstrated with the block diagram in Figure 3. The radar system also assumes that all possible communications users are transmitting at any given time; this would correspond to the maximum possible communications interference. Based on this information, we first set the radar OLFM waveform duration equal to the symbol duration, set $T_{r}$ = $T_{c}$. Then, since both systems use OLFM waveforms, and the maximum frequency used in the system is *B*, the radar can easily determine that the maximum number of possible communications users is *M* = $\left\lfloor T_{r}B/2\right\rfloor$. Use of the CoWS design by both systems and Equation (25) also provide the information that the users’ FM rates are $b_{1},\ldots ,b_{M}$; also, by design, $b_{1}$ < $b_{2}$ < … < $b_{M}$.

In order to design the FM rate of the radar OLFM waveform, we can consider increasing the range resolution for tracking the position of a target or minimizing the interference between the two systems. As the range resolution is given by:

(28) $$\sigma_{r}=\frac{c_{0}}{4\;b_{r}T_{r}}\;,$$

where $c_{0}$ is the wave velocity for electromagnetic propagation in free space, then since $T_{r}$ = $T_{c}$ is fixed, the only way to maximize $\sigma_{r}$ is to maximize the FM rate $b_{r}$. The maximum possible value is $b_{M}$, which is already taken by a communications user. However, we can select $b_{r}$ = $-b_{M}$ to also improve the range resolution. As we show next, this also reduces the interference between the two systems.

**Figure 4:**
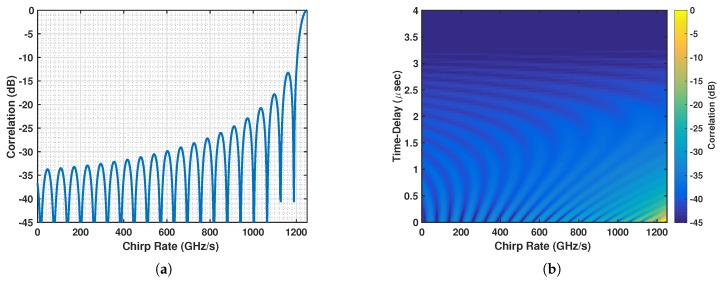
(**a**) Plot of correlation function in Equation (29) as a function of the radar FM rate using $b_{m}$ = $b_{M}$ = $B/(2T_{r})$ and (**a**) τ = 0, (**b**) varying τ.

**Figure 5:**
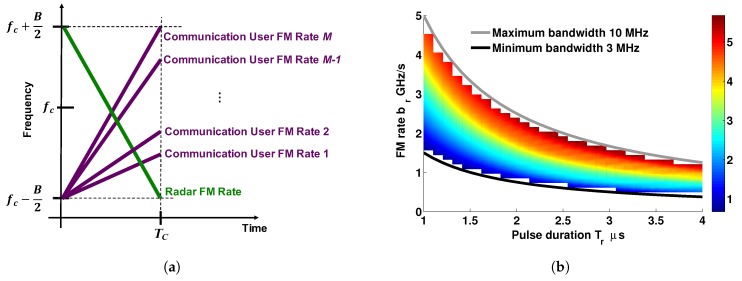
(**a**) Transmit waveform design scheme for coexisting radar and communications systems. (**b**) Achievable radar OLFM waveform parameter set for minimizing the correlation in Equation (30).

The correlation between a shifted radar LFM waveform with FM rate $b_{r}$ and the OLFM waveform used by the *m*th communication user is given by:

(29) $$\Gamma \left(b_{r},b_{m},\tau \right)={\left|\int_{0}^{T_{r}}s_{r}\left(t-\tau ;b_{r}\right)\;s_{c,m}^{*}\left(t;b_{m}\right)\;dt\right|}^{2}={\left|\int_{0}^{T_{r}}2\sqrt{t(t-\tau )}\;e^{j2\pi b_{r}{\left(t-\tau \right)}^{2}}\;e^{-j2\pi b_{m}t^{2}}\;dt\right|}^{2}\;,$$

where $b_{m}$ is given in Equation (25), *m* = 1,…,M, and $T_{r}$ = $T_{c}$. This correlation term must be minimized to reduce the interference between the two systems. As the minimization cannot be computed in closed form, we evaluated it numerically. Figure 4a shows the correlation as a function of the radar FM rate $b_{r}$ using *B* = 10 MHz, $T_{r}$ = 4 μs, τ = 0, and the highest FM rate, $b_{M}$ = $B/(2T_{r})$ = 1.25 GHz, that corresponds to user *m* = *M*. As can be seen, the radar FM rate that minimizes the correlation corresponds to the negative of the highest FM rate [40]; that is, the optimal radar FM rate is $b_{r}$ = $-b_{M}$. A similar result is obtained when the time-delay τ is allowed to vary, as shown in Figure 4b. A depiction of the resulting coexisting scheme in the time-frequency plane is shown in Figure 5a.

#### 4.2.2. Approach I-B: Radar Has Knowledge of Communications User Parameter Set Ψ_c_

**Figure 6:**
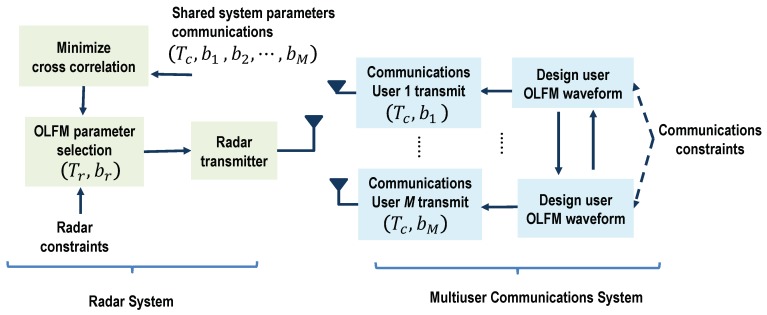
Block diagram overview of the coexistence waveform design Approach I-B: The radar receiver has knowledge of the OLFM waveform parameters, $\left\{T_{c},\;b_{1},\ldots ,b_{M}\right\}$, used by the *M* communications users.

If we assume that the radar system has knowledge of the optimal communications signal parameter set $\Psi_{c}$ in Equation (27) for $N_{c}$ ≤ *M* active communications users, then the radar waveform design can be further improved. This is demonstrated with the block diagram in Figure 6. Using this knowledge, the radar OLFM waveform parameter set $\Psi_{r}$ = $\left\{T_{r},\;b_{r}\right\}$ can be designed to minimize the communications interference ${\tilde{X}}_{c}\left(\Psi_{c},\Psi_{r}\right)$ in Equation (19) over the radar CPI. This interference component can be written as:

(30) $${\tilde{X}}_{c}\left(\Psi_{c},T_{r},b_{r}\right)=F_{\nu }\;X_{c}^{H}\left(\Psi_{c}\right)\;S_{\tau }\left(T_{r},b_{r}\right)\;,$$

to emphasize its dependence on both the communications OLFM waveform parameters obtained by minimizing the MAI and the radar OFLM waveform parameters. Note that Equation (30) assumes that the actual communication users are transmitting continuously. The communications interference ${\tilde{X}}_{c}\left(\Psi_{c},T_{r},b_{r},\right)$ can be minimized over all feasible radar OLFM waveform parameters that satisfy the following conditions:

(31) $$T_{\min}\leq T_{r}\leq T_{\max}\;\mathrm{and}\;\frac{B_{\min}}{2T_{r}}\leq b_{r}\leq \frac{B_{\max}}{2T_{r}}\;,$$

where $\left\{T_{\min},T_{\max}\right\}$ and $\left\{B_{\min},B_{\max}\right\}$ are the values of the minimum and maximum duration and bandwidth, respectively, of the radar transmit waveform. The minimum bandwidth, for example, can be obtained as $B_{\min}$ = $2b_{r}T_{r}$ for $T_{r}$ = $T_{\min}$. The optimal radar OLFM waveform parameter set is thus obtained as:

(32) $${\hat{\Psi }}_{r}=\left\{{\hat{T}}_{r},{\hat{b}}_{r}\right\}=\arg\max_{\left(b_{r},T_{r}\right)\in \Psi_{r}}\left\{\mathrm{tr}\left(\;{\tilde{X}}_{c}^{H}\left(\Psi_{c},T_{r},b_{r}\right)\;{\tilde{X}}_{c}\left(\Psi_{c},T_{r},b_{r}\right)\;\right)\right\}\;,$$

where tr(·) denotes the trace of a matrix. Note that as the term minimized is the interference between radar and communications systems, the optimized radar parameter set ${\hat{\Psi }}_{r}$ affects the performance of both systems in reducing interference. An example of feasible radar parameters ${\hat{\Psi }}_{r}$ is demonstrated in Figure 5b using $T_{\min}$ = 1 μs, $T_{\max}$ = 4 μs, $B_{\min}$ = 3 MHz, and $B_{\max}$ = 10 MHz.

### 4.3. Approach II: Coexistence Waveform Design Approach by Multiobjective Optimization

In Approach I, the radar transmit waveform was designed only based on minimizing the interference between the coexisting systems. The design did not incorporate the minimization of the MSE for estimating range and velocity of a target, even though this is a very important performance metric for radar systems. Whereas minimizing the interference is a joint system performance metric, the MSE is only a radar specific performance metric. However, both of these metrics affect the transmit radar OLFM waveform parameter set $\Psi_{r}$. Although not necessarily conflicting objectives, the two performance metrics may need to be traded off, depending on the expected system outcome. For example, the bandwidth of the radar waveform can be chosen to be large for a higher range resolution, as then the MSE of the time delay estimate is decreased. However, it is not known how increasing the radar waveform bandwidth can affect the interference between the two systems.

**Figure 7:**
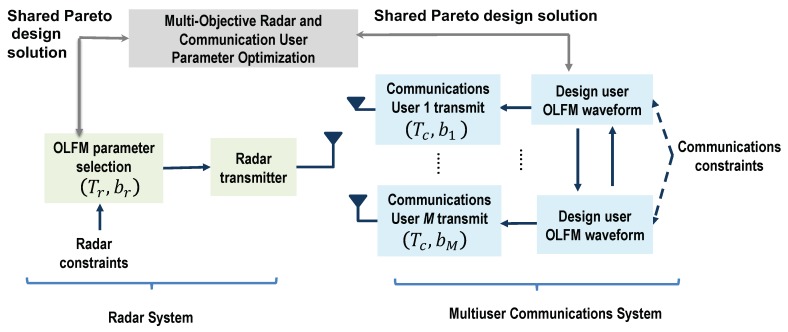
Block diagram overview of coexistence waveform design Approach II: Pareto optimization is used to jointly optimize the OLFM waveform parameters of both systems; the designed parameters are relayed to both the radar and communications receivers.

The coexisting waveform design optimization is thus formulated as a multiobjective or Pareto optimization in order to obtain a set of acceptable tradeoff optimal solutions [42,43]. Figure 7 provides an overview of this joint waveform system design. In particular, we considered an optimization problem over a waveform parameter vector g with L≥2 objective functions denoted by $\zeta_{l}\left(g\right)$, *l* = 1,…L. We also considered a set of waveforms G whose parameter vectors g can be used by both systems. The multiobjective optimization can then be written as [42]:

$$\tilde{g}=\min_{g\in G}\;\;{\left[\zeta_{1}\left(g\right)\;\;\ldots \;\;\zeta_{L}\left(g\right)\right]}^{T}\;.$$

For the coexistence radar and communications problem, the waveform parameter vector is given by g = ${\left[\Psi_{c}\;b_{r}\;T_{r}\right]}^{T}$. It consists of the radar OLFM waveform parameters $b_{r}$ and $T_{r}$, constrained using Equation (31), and the communications user OLFM waveform parameters $\Psi_{c}$ = $\left\{{\hat{T}}_{c},{\hat{b}}_{1},\ldots ,{\hat{b}}_{N_{c}}\right\}$ in Equation (27). Thus, for the communication system, the OLFM waveform parameters are already optimized to reduce MAI between users using Equation (6) for $N_{c}$ ≤ *M*. The overall waveform set G thus consists of OLFM waveforms that satisfy Equations (6) and (31) for communications and radar, respectively. Although different objective functions for each system can be selected, we concentrated on using the radar time-delay estimation MSE and the system coexistence interference. In particular, we selected the radar time-delay estimation MSE, denoted by $\zeta_{1}\left({\left[\Psi_{c}\;b_{r}\;T_{r}\right]}^{T}\right])$, and the interference between the radar and communications waveforms, denoted by $\zeta_{2}\left({\left[\Psi_{c}\;b_{r}\;T_{r}\right]}^{T}\right])$.

The computation of the joint optimization approach was performed by sampling the boundaries associated with the feasible regions of the parameters of the radar waveform and the communications users waveforms. The complexity of computing the correlation between system waveforms is largely dominated by a dense matrix product whose order of magnitude is $O(N_{\nu }N_{\tau }^{2})$; here, $N_{\tau }$ and $N_{\nu }$ are the total number of range and Doppler bins, respectively, used for processing at the radar receiver in Section 3.2.2. The MSE is obtained by implementing the Slepian–Bangs formulation normally used to compute Cramér–Rao lower bounds on the estimation variance; the complexity is in the order of magnitude $O(N_{s}^{2})$, where $N_{s}$ in Equation (9) is the number of discrete-time samples of the radar waveform, which depends on the prescribed sampling rate.

## 5. Simulations Results

### 5.1. Approach I-A Simulation

**Figure 8:**
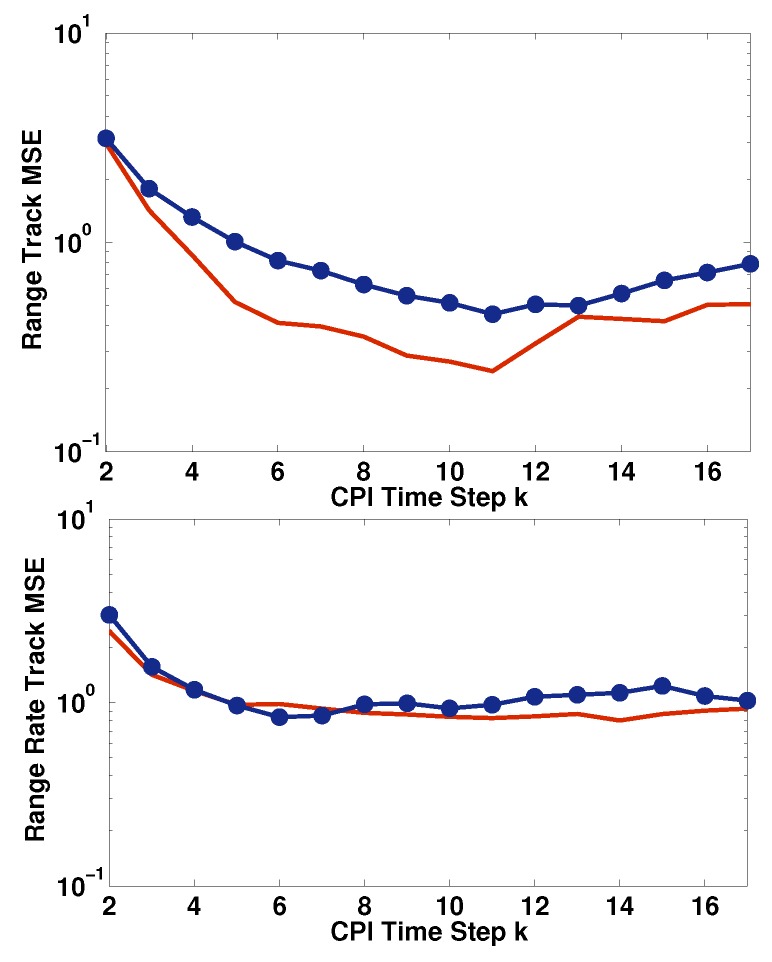
MSE for range (**top**) and range–rate (**bottom**) estimation when the radar OLFM waveform FM rate is $-b_{20}$ (**red**) and $-b_{1}$ (**blue**) for three users at −18 dB SINR at the radar receiver.

The first simulation demonstrates the design of the radar OLFM waveform parameters to minimize the interference in Equation (29) between the coexisting systems using waveforms with the same duration (see Approach I-A in Section 4.2.1). As discussed in Section 4.2, the target’s range resolution in Equation (28) can be improved by selecting the FM rate of the radar OLFM waveform equal to $-b_{M}$. In this simulation, *M* = 20 and $N_{c}$ = 3, $T_{c}$ = $T_{r}$ = 4 μs, *B* = 10 MHz. The OLFM waveform FM rates selected for the three users are $b_{18}$, $b_{19}$, and $b_{20}$, following the notation in Equation (25) for minimizing MAI. The SINR at the radar receiver is −18 dB. As can be seen in Figure 8, when the radar OLFM waveform FM rate is selected to be $-b_{20}$ = $-b_{M}$, the MSE for range estimation is lower than when the FM rate is selected to be $-b_{1}$.

The remaining simulations, which were used to demonstrate Approach I-B and Approach II, used the following common parameters. An MU AWGN communications channel was used with $N_{c}$ = 4 active communications users, each employing a 16-PSK modulation. The required gross bit rate is *R* = 1 Mbps, resulting in a symbol duration $T_{c}$ = 4 μs. The allocated bandwidth available to the radar and the communications systems is *B* = 10 MHz. The pulse–Doppler radar uses *K* = 50 pulses per CPI, and it is operating at a constant 10 kHz pulse repetition frequency (PRF).

### 5.2. Approach I-B Simulation

Following the optimization procedure of Section 4.2.2, we initially computed the optimal selection of communications users FM rates from Equation (27) that satisfy Equation (6) given $N_{c}$ = 4. The optimization parameters for the radar in Equation (31) are constrained such that $1\leq T_{r}\leq 4$ μs and the minimum and maximum bandwidth of the radar waveform are $3\leq 2b_{r}T_{r}\leq 10$ MHz. A plot of the feasible radar parameters $\Psi_{r}$ and their associated cost value from Equation (30) using the optimized selection of communications users are demonstrated in Figure 5b.

**Figure 9:**
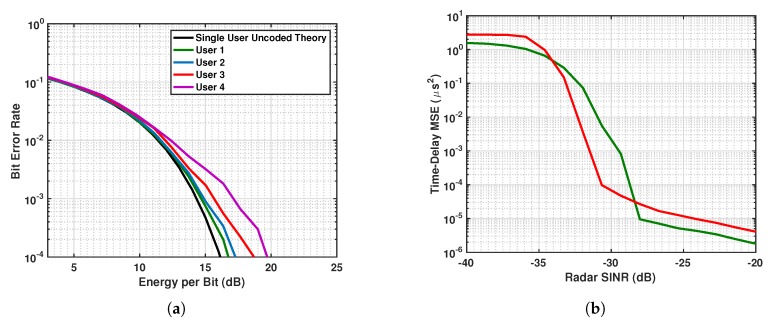
(**a**) BER and (**b**) time-delay estimation mean-squared error (MSE) performance (**red**) for coexisting systems using communications parameters $\Psi_{c}$ in Equation (27) and radar parameters $\Psi_{r}$ in Equation (32). In (**b**), also shown (**green**) is the MSE performance obtained using a radar waveform that minimizes the MSE.

The BER performance for the users is demonstrated in Figure 9a as a function of energy per bit for the case where the radar waveform is chosen to minimize system interference. The corresponding time-delay estimation MSE obtained by the radar waveform that minimizes the correlation in Equation (32) is shown in Figure 9b in red. This is compared to the MSE obtained by a radar waveform that directly minimizes the MSE (green). As can be seen, the radar waveform that minimizes system interference does not, in general, provide the best MSE performance for the radar. As a result, we utilized the multiobjective waveform approach to derive radar parameters that jointly optimize both the radar system and communications system performance.

### 5.3. Approach II Simulation

**Figure 10:**
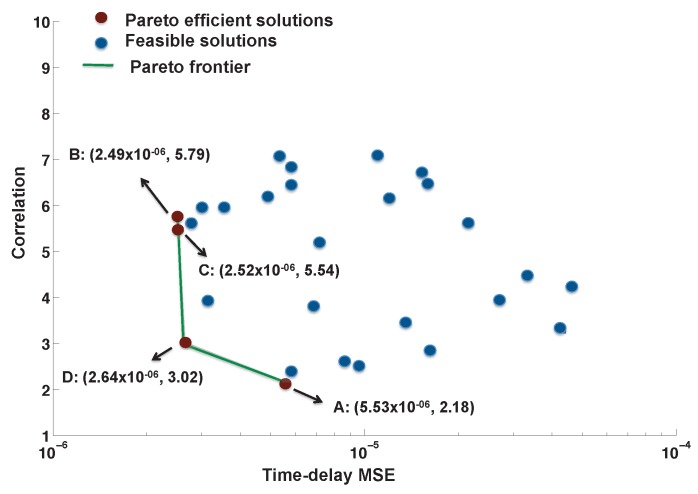
The possible solutions obtained by minimizing both the time-delay estimation MSE and the system correlation are shown in blue; the efficient Pareto solutions (shown in maroon) are connected (using green) with the Pareto frontier.

In Approach I, it was demonstrated that the interference between radar and communications systems is reduced at the cost of decreasing radar system performance in terms of MSE. This approach aims to use Pareto optimization to obtain parameters that are jointly optimal for both the communications and radar system waveform parameters. The feasible parameter vectors that correspond to the objective functions of time-delay estimation MSE and interference between the two systems appear as two-dimensional points in Figure 10. The points were obtained using the conditions in Equations (6) and (31), and the same system parameters were utilized as in Approach I-B. The optimal communications FM rates in $\Psi_{c}$ were obtained by employing simulated annealing as discussed in Section 4.1 and satisfy Equations (6) and (27). For the radar parameter conditions in Equation (31), the waveform bandwidth is constrained between 3 and 10 MHz, and the radar pulse duration is between 1 to 4 μs. The Pareto frontier (in green) in Figure 10 connects the efficient solutions of the Pareto maximization. Efficient solutions are those that are not dominated by other possible outcomes in the optimization problem.

**Figure 11:**
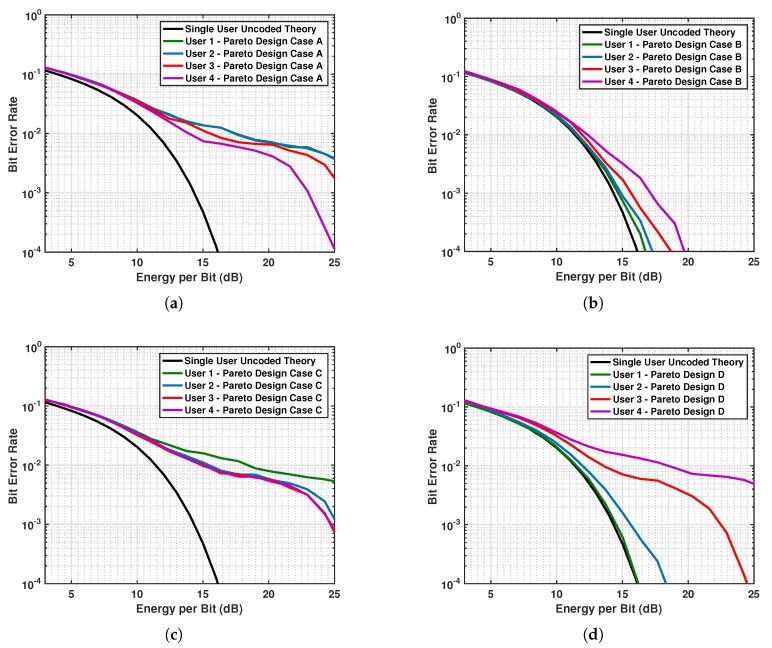
Four communications users BER performance using the Pareto designs: (**a**) A; (**b**) B; (**c**) C; and (**d**) D.

**Figure 12:**
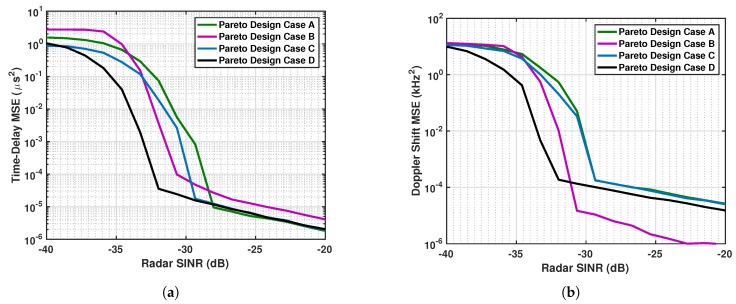
(**a**) Time-delay MSE and (**b**) Doppler estimation MSE performance for the Pareto designs that correspond to the four Pareto frontier ponts in Figure 10.

The efficient points on the Pareto frontier obtained from the simulated feasible vectors are labeled as A, B, C, D and shown in maroon in Figure 10. These points correspond to OLFM waveforms whose radar parameters $\left({\tilde{b}}_{r},{\tilde{T}}_{r}\right)$ are optimal. The coordinates of these points provide the time-delay estimation MSE and the system correlation. The minimum correlation is achieved by point A, and the miminum time delay estimate MSE is achieved by point B. The coordinates of points C and D have time-delay estimate MSEs that are close in value. We simulated both systems using the OLFM waveform radar parameters that resulted from the four Pareto efficient points. The four communications users’ BER performance is shown in Figure 11a–d for each Pareto design. The figure shows that Design B yields the best BER performance for all four users; this is because point A was used in Figure 10 that corresponds to the minimum correlation value. Figure 12 demonstrates the estimation MSE for both time delay and Doppler. This figure shows that Pareto Design A provides the minimum time delay estimation MSE at high SINR. This follows because the point used from Figure 10 is point B, the point of minimum MSE. The tradeoff between joint system performance and design selection can be seen by the fact that the design that provides the best BER performance (Design B) does not provide the best MSE estimation performance; the MSE performance of Design B is slightly worse than for the other designs at high SINR. Note that the Pareto optimal points that lie on the frontier are only optimal in the sense that they provide the best tradeoff performance when used to design the transmit system waveforms. The selected waveforms are, however, suboptimal, as they do not simultaneously result in both minimum system correlation and minimum radar MSE estimation performance.

## 6. Conclusions

In this paper, we considered a pulse–Doppler radar and multiuser wireless communications systems that share the same spectrum. We proposed a joint transmit waveform with the same time-varying amplitude and quadratic phase functions, and we developed a design method to select waveform parameters by optimizing joint system performance metrics. Using multiobjective optimization, the tradeoff in reducing both overall interference and state parameter estimation error was demonstrated. Note that the designed parameters only include the frequency-modulation rate and duration of the waveform but not the waveform’s phase function. It may be possible to improve the aforementioned performance tradeoff when the time-varying phase function is also used in the design, especially at varying output SNR values. As we demonstrated in References [17,21] using a deviation measure of the Barankin bound from the Cramér–Rao lower bound on the estimated target state covariance, waveforms with a logarithmic phase function result in lower MSE than waveforms with a quadratic phase function.
